# Sexual orientation disparities in physical health: age and gender effects in a population-based study

**DOI:** 10.1007/s00127-015-1116-0

**Published:** 2015-08-23

**Authors:** Richard Bränström, Mark L. Hatzenbuehler, John E. Pachankis

**Affiliations:** Department of Clinical Neuroscience, Karolinska Institutet, Berzelius väg 3, 171 77 Stockholm, Sweden; Department of Sociomedical Sciences, Columbia University, New York, NY USA; Chronic Disease Epidemiology, Yale School of Public Health, New Haven, CT USA

**Keywords:** Self-rated health, Minority stress, Health behaviors, Gay/bisexual, Sexual orientation, Life span

## Abstract

**Background:**

Recent studies have identified substantial health disparities between lesbian, gay, and bisexual (LGB) individuals compared to heterosexuals. However, possible variation in sexual orientation health disparities by age and according to gender remains largely unexplored.

**Purpose:**

To examine physical health disparities between LGB and heterosexual individuals in a general population sample in Sweden, to explore potential age and gender differences in these disparities, and to test potential mechanisms underlying any observed disparities.

**Method:**

Between 2008 and 2013, 60,922 individuals (16–84 years of age) responded to nationwide population-based health surveys. In the sample, 430 (0.7 %) individuals self-identified as gay/lesbian and 757 (1.3 %) self-identified as bisexual. Logistic and negative binomial regression analyses were used to explore health disparities based on sexual orientation.

**Results:**

Overall, LGB individuals were more likely to report worse self-rated health as well as more physical health symptoms (e.g., pain, insomnia, dermatitis, tinnitus, intestinal problems) and conditions (e.g., diabetes, asthma, high blood pressure) compared to heterosexuals. However, these physical health disparities differed by age. Disparities were largest among adolescents and young adults and generally smallest in older age groups. Health behaviors and elevated reports of exposure to perceived discrimination, victimization, and threats of violence among sexual minorities partially explained the sexual orientation disparities in physical health.

**Conclusions:**

Age emerged as an important effect modifier of physical health disparities based on sexual orientation. Gender-specific findings suggest that sexual orientation disparities persist into adulthood for women but are gradually attenuated for older age groups; in contrast, for men, these disparities disappear starting with young adults. These results support a developmental model of minority stress and physical health among LGB individuals.

## Introduction

During the past several years, public health policy and research have begun to address the substantial health disparities that exist between sexual minority [e.g., individuals who identify as lesbian, gay, and bisexual (LGB) or engage in same-sex sexual behavior] and heterosexual individuals [1]. Most of this attention has focused on mental health disparities, with population-based studies from both North America and Europe showing that LGB individuals are significantly more likely to be diagnosed with major depression and several anxiety disorders compared to heterosexual individuals and that LGB youths are at greater risk for suicide attempts than non-LGB youths [2–5]. With the exception of HIV/AIDS, much less is known about sexual orientation disparities in physical health, although a recent review identified substantial evidence of elevated reports of physical health problems among LGB, compared to heterosexual, individuals [6]. In this review, the majority of studies were from North America and showed poorer health among LGB individuals, measured both on general indices of health (e.g., self-rated health status, acute physical symptoms) [7, 8], prevalence of specific health conditions (e.g., asthma, headaches, gastro-intestinal problems) [9, 10], and risk of disease (e.g., cardiovascular disease, cancer) [11, 12], as compared to heterosexuals. Despite accumulating evidence for the existence of sexual orientation health disparities, studies typically rely on data with several limitations, including small convenience and non-representative samples, cross-sectional data, self-report measures of physical health, and specific age groups.

Sexual orientation health disparities have largely been explained through minority stress theory, which describes the excess stress that LGB individuals experience compared to heterosexual individuals by virtue of their stigmatized sexual orientation [2]. This minority stress, in the form of prejudice, discrimination, sexual orientation concealment, expectations of rejection, and internalized stigma [13], additively combines with general life stress to confer adverse health outcomes. In fact, LGB individuals report more stressors and fewer coping resources compared to heterosexuals [13]. This increased stress exposure at least partly accounts for sexual orientation disparities in mental and physical health [6, 14].

Given the unique forms of stress experienced by sexual minority individuals at various developmental periods, sexual orientation disparities in physical health outcomes might differ by age. For instance, parental and peer rejection [15] and the stress of concealing and disclosing one’s sexual orientation [16] are likely to particularly affect the health of younger, compared to older, sexual minority individuals, given that younger LGB individuals are closer in time to these stressors and have less experience coping with these stressors. However, other stressors, such as workplace discrimination, family stress, social isolation, as well as prejudice and discrimination more generally, might accumulate over the life course to compromise health, consistent with the life course accumulating effects found to occur among individuals from disadvantaged socioeconomic backgrounds and racial and ethnic minorities in the US [17–19]. However, with few exceptions [7, 20, 21], existing studies treat age as a potential confounder and thus control for it in statistical analyses, rather than examining sexual orientation disparities in physical health outcomes by age. Consequently, whether sexual orientation disparities in physical health outcomes are stronger among younger or older age groups remains to be determined. In a recent study of successful aging among LGB older adults, Fredriksen-Goldsen and colleagues used a resilience framework specifying a number of general and LGB-specific risk and protective factors as contributors to self-rated health [21]. In particular, the study found that the negative effect of lifetime victimization and discrimination on physical health was strongest for the oldest age group as compared to younger old adult LGB individuals, even though the oldest age group was less likely to report such lifetime experiences. The authors conclude that these findings might be a consequence of historic social contexts that demonstrate a cohort effect, where concealment of sexual orientation might have been protective against exposure to victimization and discrimination, but simultaneously increased vulnerability to the negative consequences of such experiences [21]. Their findings highlight the importance of investigating and identifying factors leading to positive health outcomes among LGB individuals and exploring age group variations in such factors.

Further, although sexual orientation health disparities have been documented for both men and women, recent studies have uncovered gender differences in these disparities. For example, greater prevalence of obesity and other risks factors for cardiovascular disease have been found for lesbian compared to heterosexual women but not for gay compared to heterosexual men [11]. Additionally, elevated risk for common health conditions and health limitations have been found for sexual minority women compared to heterosexual women and elevated health concerns related to HIV infection are found among sexual minority men compared to heterosexual men [22]. Elevated rates of arthritis and asthma exist for lesbian/bisexual women but not for gay/bisexual men [8, 20]. However, studies examining gender differences in sexual orientation disparities have not examined gender differences as a function of age, suggesting the importance of examining age patterns in sexual orientation physical health disparities for men and women separately.

The aim of the current study was twofold: (1) to examine physical health disparities between sexual minority individuals and heterosexuals in a general population sample in Sweden and (2) to explore potential gender and age differences in such disparities. We also examined measures of self-reported exposure to stressors consistent with minority stress theory (e.g., perceived discrimination, victimization, threats of violence) and self-reported health-risk behaviors (e.g., smoking, alcohol consumption, physical activity) and Body Mass Index (BMI), which allows for testing potential mechanisms underlying any observed disparities across age groups. The study is based on a sample from Sweden, a country with a low level of legal and administrative discrimination against sexual minorities as well as high social acceptance of sexual minorities as compared to other countries [23]. Further, the universal health care system in Sweden eliminates potential confounding due to sexual orientation differences in health care access, which has been observed in the US [24, 25].

## Methods

### Study sample

Between 2008 and 2013, yearly nationwide population-based health survey studies were conducted in unrestricted random samples (*n* = 20,000 per year) of the population in Sweden, 16–84 years of age, by the Swedish National Institute of Public Health. A total of 60,922 individuals responded to the survey via paper-and-pencil mailed questionnaires or self-administered web surveys. The overall response rate was between 48.8 and 55.7 % each year, and it was higher among women and in the older age groups. To adjust the results for varying response rates, post-stratification weights were used to compensate for lower response rates in some groups, making the sample representative for the total population. In addition to a question regarding sexual orientation, the survey included questions covering a number of factors relating to socio-demographic background, health status, and health determinants, and was supplemented with data from administrative national registries regarding income and ethnicity. The study was approved by the Regional Ethics Committee in Stockholm (No. 2013/2200-31/2).

### Measures

#### Sexual orientation

Individuals were classified based on self-identification of sexual orientation using the following item: “What is your sexual orientation?” with the response categories: “heterosexual,” “bisexual,” “homosexual,” and “not sure.” The response rate for this question was between 92.9 and 95.4 % across years, with 430 (0.7 %) individuals self-identifying as gay/lesbian and 757 (1.3 %) self-identifying as bisexual. We excluded 980 (1.6 %) individuals who responded that they were uncertain of their sexual orientation, as previous studies have shown that this group often consists of a heterogeneous mix of respondents in terms of sexual identity [26]. While some people do not know their sexual orientation because they are undecided, studies have indicated that the majority of people who choose such responses in population surveys are doing so because they did not understand the question [27]. Those who responded that they were “not sure” of their sexual orientation did not differ significantly in age from heterosexuals, but were more often men, born outside of Sweden, had lower income, were less often married or partnered, and were more likely to report poor general health as compared to those reporting being heterosexual.

#### Physical health outcome variables

We examined two physical health outcomes: (1) self-rated general health and (2) number of physical symptoms. Self-rated health was assessed with the following item: “How would you rate you general health?” and response options included: very good, good, fair, poor, very poor. Consistent with prior research, we created a dichotomous variable comparing individuals with fair, poor, or very poor health versus those reporting very good or good health [28]. Prior research has demonstrated that self-rated health is a valid indicator of health status and/or the presence of disease and predicts mortality risk [29].

The number of physical symptoms was assessed with two items: “Do you currently have any of the following problems or symptoms?” and “Do you have any of the following conditions?” The checklist of 10 problems/symptoms included: pain in neck, back pain, headache, pain in hand/arm/legs, fatigue, insomnia, dermatitis, tinnitus, urinary incontinence, and intestinal problems. The checklist of current chronic physical conditions included: diabetes, asthma, allergy, and high blood pressure. A count variable of these 14 items was created, and respondents were categorized into a dichotomous variable where individuals were identified as either having an elevated number of physical symptoms or conditions [i.e., >5 symptoms/conditions (cut-off for upper quartile of number of symptoms)], or not having an elevated number of symptoms or conditions.

#### Covariates

Four classes of control variables relating to socio-demographics, experiences of minority stress, health-risk behaviors, and body mass index (BMI) were included. Socio-demographic factors included yearly household income, ethnicity (nation of birth categorized into groups of geographic regions), and urbanicity (living in larger city, smaller city, or rural community), which were collected from national registries and linked to the questionnaire data, as well as self-reported relationship status (living with partner versus single).

Minority stress experiences were assessed as self-reported exposure to perceived discrimination during the past three months (“During the past three months, have you been treated in a way that made you feel discriminated against?”), victimization during the past 12 months (“During the past 12 months, have you been exposed to physical violence?”), and threats of violence during the past 12 months (“During the past 12 months, have you been exposed to a threat or threats of violence in a way that made you frightened?”).

Health-risk behaviors included: tobacco use, use of alcohol, and frequency of physical activity. The question regarding smoking was used to categorize the respondents into current daily smokers versus non-smokers. Two different measurements were used to describe the respondents’ use of alcohol. The first concerned frequency of heavy drinking during the past 12 months, based on one question regarding frequency of intensive alcohol consumption (defined as drinking at least one bottle of wine or equivalent during one occasion). The second measure concerned total weekly amount of alcohol consumed, which was categorized into risk consumers and non-risk consumers. Male respondents were categorized as risk consumers of alcohol if they reported an average weekly consumption of more than 14 drinks and women if they reported an average weekly consumption of more than nine drinks, in accordance with the threshold for hazardous weekly alcohol consumption proposed by the Swedish National Institute of Public Health [30]. Physical activity was assessed using a single-item measure of current frequency of weekly physical activity (i.e., at least moderately intense physical activity) with response alternatives in five categories. Based on their responses, participants were categorized into three categories: physically inactive (less than 60 min/week), moderately physically active (60–180 min/week), and physically active (more than 180 min/week). The categorization was based on the global recommendation of levels of physical activity presented by the World Health Organization [31]. Further, the participants were asked to report height and weight, used to calculate body mass index (BMI). The BMI variable was calculated by dividing participant weight in kilograms by their squared height in centimeters, and was used as a continuous variable and to categorize individuals into normal weight/underweight (BMI < 25) and overweight/obese (BMI ≥ 25).

### Statistical analysis

After examining descriptive statistics of participants’ responses by socio-demographic characteristics, we examined differences based on sexual orientation in physical health outcomes, stratifying by gender and age. Logistic and negative binomial regressions were used to estimate sexual orientation-related differences in self-rated general heath and number of physical symptoms and conditions. The analyses were adjusted for a number of covariates entered in three separate sets: (1) demographic characteristics (income, ethnicity, relationship status, and urbanicity); (2) health behavior variables and BMI; and (3) potential mediating variables (perceived discrimination, victimization, and threat of violence). In all analyses, post-stratification weights were used to adjust for selection probabilities and non-response. For the purpose of comparing change in the estimate for the sexual orientation disparity in self-rated general health when new variables were entered into the analyses, standardization was used to make coefficients comparable across models. To standardize estimates, coefficients were divided with the estimated standard deviation (y-standardization) as described by Mood [32] and change in percentages were calculated between models using these standardized estimates. To examine age effects, we categorized participants into four age groups. Due to the low number of LGB respondents in the oldest age category (i.e., 65–84), and for the purpose of having sufficient number of LGB respondents in all age categorizes, the oldest age groups were collapsed into one (46–84 years). To statistically test the effect of age on sexual orientation-related health disparities, we preformed regression analyses entering variables for sexual orientation and age groups, as well as the interaction term for those variables (sexual orientation × age group). All analyses were performed using SPSS version 22.

## Results

**Fig. 1:**
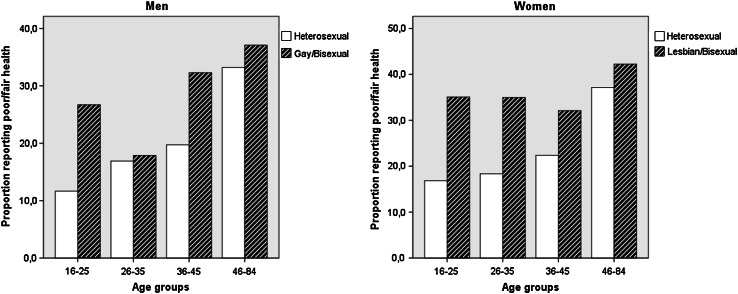
Proportion of men and women reporting poor/fair health by sexual orientation showing differences by age group

Table 1 presents demographic characteristics, exposure to stressful events, and health behaviors by sexual orientation separately for men and women. Among both men and women, the sexual orientation groups differed on all demographic variables. The sexual minority groups were more likely to live in larger cities, have lower income, be non-Swedish born, be younger, and were less likely to live with a partner. LGB respondents were more likely to report exposure to stressful life events. Gay and bisexual men were more likely to engage in all health-risk behaviors than heterosexual men. Lesbian and bisexual women were more likely to report risk consumption of alcohol, and binge drinking of alcohol than heterosexual women, but there were no group differences in physical activity. In Table 2, associations of self-reported health, physical symptoms, discrimination, victimization, and threats of violence, with sexual orientation, age, and sexual orientation × age interactions are presented. All sexual orientation × age interactions were significant both among men and women, except for threats of violence among men. The interactions showed decreasing disparities with increasing age for all variables except reported victimization among men. The difference in reported victimization between LGB and heterosexual men were much larger in the oldest age group. The interaction for self-rated health is illustrated in Fig. 1. Based on the results of these interaction analyses, age-stratified models are presented below.

**Table 1:** Sample characteristics, measures of physical health, exposure to stressful events, health behaviors, and BMI by gender and sexual orientation

	Men				Women			
	Self-identified gay, *n* = 257	Self-identified bisexual, *n* = 274	Self-identified heterosexual, *n* = 25 069		Self-identified Lesbian, *n* = 173	Self-identified Bisexual, *n* = 483	Self-identified Heterosexual, *n* = 30 246	
Age (years)	*n* (%)	*n* (%)	*n* (%)		*n* (%)	*n* (%)	*n* (%)	
16–25	32 (12.5)	62 (22.6)	2635 (10.5)	F^a^ = 35.73***	29 (16.8)	157 (32.5)	3449 (11.4)	F^b^ = 171.56***
26–35	51 (19.8)	36 (13.1)	2828 (11.3)		46 (26.6)	134 (27.7)	3841 (12.7)	
36–45	67 (26.1)	40 (14.6)	3840 (15.3)		41 (23.7)	87 (18.0)	4904 (16.2)	
46–55	34 (13.2)	51 (18.6)	4187 (16.7)		22 (12.7)	43 (8.9)	5346 (17.7)	
56–65	42 (16.3)	36 (13.1)	5241 (20.9)		19 (11.0)	38 (7.9)	5862 (19.4)	
66–75	21 (8.2)	25 (9.1)	4427 (17.7)		10 (5.8)	18 (3.7)	4714 (15.6)	
76–84	10 (3.9)	24 (8.8)	1911 (7.6)		6 (3.5)	6 (1.2)	2130 (7.0)	
Household income	M (SD)^c^	M (SD)^c^	M (SD)^c^		M (SD)^c^	M (SD)^c^	M (SD)^c^	
Mean yearly income in tSEK^d^	342 (518)	367 (282)	408 (389)	F = 6.22**	328 (245)	312 (229)	389 (514)	F = 7.08**
Household status	%^e^	%^e^	%^e^		%^e^	%^e^	%^e^	
Living with partner	45.7	33.8	65.3	F = 99.78***	59.6	52.6	63.7	F = 14.28***
Urbanity	%^e^	%^e^	%^e^		%^e^	%^e^	%^e^	
Larger city	58.5	43.6	32.5	F = 59.10***	53.2	41.1	33.3	F = 16.29***
Smaller city	26.1	32.5	34.2		24.6	29.3	34.1	
Rural community	15.4	24.0	33.3		22.2	29.6	32.6	
Nation of birth	%^e^	%^e^	%^e^		%^e^	%^e^	%^e^	
Sweden	70.6	76.7	86.0	F = 25.73***	76.4	79.4	85.2	F = 5.22**
Other Nordic Country	5.9	2.1	3.2		4.9	2.8	3.9	
Other European Country	11.9	11.0	5.2		9.7	11.2	5.3	
North/South America	3.3	2.1	1.1		4.2	2.3	1.3	
Asia	8.2	6.0	3.6		4.2	4.1	3.7	
Africa	0.0	2.1	0.9		0.7	0.2	0.6	
Self-rated general health	%^e^	%^e^	%^e^		%^e^	%^e^	%^e^	
Fair or poor physical health	28.4	31.1	34.7	F = 4.83**	27.5	38.5	28.6	F = 12.30***
Physical Health symptoms	M (SD)^c^	M (SD)^c^	M (SD)^c^		M (SD)^c^	M (SD)^c^	M (SD)^c^	
Number of physical symptoms and conditions (0–14)	3.8 (2.7)	3.7 (2.6)	3.2 (2.4)	F^g^ = 16.00***	4.1 (2.4)	4.6 (2.5)	4.1 (2.6)	F^h^ = 10.06***
Exposure to stressful events	%^e^	%^e^	%^e^		%^e^	%^e^	%^e^	
Discrimination, past 3 months	31.9	29.4	15.9	F = 53.49***	33.7	44.4	23.3	F = 65.44***
Victimization in the past 12 months	3.7	5.9	3.5	F = 2.66	1.9	7.1	2.2	F = 26.83***
Threat of violence, past 12 months	8.3	7.0	3.7	F = 14.56***	9.9	12.3	4.2	F = 45.71***
Health Behaviors	%^e^	%^e^	%^e^		%^e^	%^e^	%^e^	
Smoking	22.4	22.1	10.4	F = 49.44***	9.1	17.9	12.7	F = 7.24**
Risk consumption of alcohol	23.9	21.0	16.5	F = 8.76***	16.6	21.5	9.8	F = 43.21***
Binge drinking of alcohol	28.8	24.8	22.5	F = 4.04*	14.2	15.6	7.9	F = 24.82***
Physical activity				F = 13.46***				F = 0.33
Physically inactive	6.9	8.8	5.1		9.9	5.6	4.9	
Moderate physical activity	49.4	41.9	39.5		34.5	42.1	41.8	
Physically active	43.7	49.3	55.4		55.6	52.3	53.3	
Body mass index (BMI)	M (SD)^c^	M (SD)^c^	M (SD)^c^		M (SD)^c^	M (SD)^c^	M (SD)^c^	
Mean BMI	24.8 (4.4)	25.7 (5.6)	25.9 (4.0)	F = 12.38***	24.9 (5.2)	24.3 (5.0)	24.9 (4.7)	F = 3.71*

* Significant at *p* < 0.05; ** significant at *p* < 0.01; *** significant at *p* < 0.001

^a^Mean age for gay (mean = 41.2, SD = 15.4) and bisexual (mean = 39.8, SD = 18.0) men were significantly lower than for heterosexual men (mean = 46.3, SD = 17.9)

^b^Bisexual women were significantly younger (mean = 38.2, SD = 15.5) than lesbian (mean = 33.3, SD = 13.9) and heterosexual women (mean = 47.2, SD = 18.1), and lesbians were significantly younger than heterosexual women

^c^Weighted means and standard errors

^d^Swedish kronor, in thousands

^e^Weighted percentages

**Table 2:** Logistic regressions with associations between self-reported health, physical symptoms, discrimination, victimization, and threats of violence, with sexual orientation, age, and sexual orientation × age interactions

	Poor/fair self-rated health		No. of physical symptoms (>5)		Discrimination		Victimization		Threats of violence	
	AOR^a^ (95 % CI)		AOR^a^ (95 % CI)		AOR^a^ (95 % CI)		AOR^a^ (95 % CI)		AOR^a^ (95 % CI)	
Men										
Sexual orientation (SO)										
Heterosexual	1		1		1		1		1	
Gay/bisexual	2.99***	(2.07–4.32)	3.28***	(2.13–5.04)	2.99***	(2.16–4.13)	0.76	(0.41–1.40)	1.00	(0.55–1.80)
Age										
16–25	1		1		1		1		1	
26–35	5.06***	(2.70–9.52)	3.73***	(1.79–7.77)	1.44	(0.85–2.45)	1.48	(0.34–6.41)	0.34*	(0.13–0.84)
36––45	4.38***	(2.52–7.60)	4.13***	(2.19–7.78)	1.81*	(1.07–3.06)	0.21**	(0.07–0.61)	0.22**	(0.09–0.55)
46–84	14.29***	(8.72–23.42)	11.82***	(6.70–20.83)	1.45	(0.88–2.39)	0.05***	(0.02–0.13)	0.22**	(0.09–0.52)
SO by Age interaction										
SO × 16–25	1		1		1		1		1	
SO × 26–35	0.32***	(0.18–0.59)	0.40**	(0.20–0.79)	0.71	(0.43–1.16)	0.40	(0.10–1.67)	1.86	(0.79–4.38)
SO × 36–45	0.54*	(0.32–0.90)	0.55*	(0.31–0.98)	0.53*	(0.33–0.87)	1.74	(0.64–4.72)	2.30	(0.99–5.34)
SO × 46–84	0.33***	(0.21–0.52)	0.40***	(0.24–0.67)	0.42***	(0.26–0.68)	3.66**	(1.55–8.64)	1.89	(0.84–4.23)

Women										
Sexual orientation (SO)										
Heterosexual	1		1		1		1		1	
Lesbian/bisexual	2.62***	(1.97–3.49)	2.22***	(1.65–2.99)	2.28***	(1.74–2.98)	2.52***	(1.58–4.04)	2.63***	(1.82–3.81)
Age										
16–25	1		1		1		1		1	
26–35	1.31	(0.82–2.09)	1.31	(0.81–2.14)	1.18	(0.77–1.82)	2.56	(0.92–7.14)	2.04	(0.99–4.17)
36–45	2.99***	(1.80–4.97)	2.26**	(1.35–3.79)	1.37	(0.84–2.23)	1.14	(0.39–3.31)	0.95	(0.45–1.99)
46–84	7.04***	(4.34–11.42)	5.14***	(3.10–8.50)	0.70	(0.42–1.18)	0.31*	(0.10–0.93)	0.41*	(0.18–0.94)
SO by Age interaction										
SO × 16–25	1		1		1		1		1	
SO × 26–35	0.86	(0.57–1.32)	0.86	(0.55–1.33)	0.77	(0.52–1.15)	0.37*	(0.14–0.94)	0.51*	(0.27–0.96)
SO × 36–45	0.56*	(0.35–0.90)	0.71	(0.44–1.15)	0.58*	(0.37–0.92)	0.66	(0.25–1.75)	0.89	(0.46–1.73)
SO × 46–84	0.44***	(0.28–0.70)	0.60*	(0.37–0.96)	0.63	(0.38–1.03)	1.16	(0.42–3.21)	1.04	(0.48–2.25)

* Significant at *p* < 0.05; ** significant at *p* < 0.01; *** significant at *p* < 0.001

^a^Adjusted odds ratios with analyses adjusted for income, ethnicity, household status, and living in urban or rural communities. All models take into account sample weights

### Self-reported general health

In unadjusted analyses, gay/bisexual men age 16–25 years and 36–45 years, and lesbian/bisexual women younger than 46 years, reported poorer health than heterosexuals (Table 3). Further, in multivariate models controlling for covariates we found a graded age relationship in sexual orientation-based disparities in self-rated health. The strongest disparity was found in the youngest age groups and disparity was attenuated with increasing age. An exception to this pattern was found for men aged 36–45 years. In this age group, the sexual orientation disparity was higher than among men aged 26–35 years, but the disparity was eliminated with inclusion of health behavior into the model (Model 3). In the fully adjusted models (Model 4), no sexual orientation differences in self-rated health were observed among men older than 25 years and women older than 35 years, and for all age groups the disparities were reduced with inclusion of covariates and the mediators. Among the age groups showing a sexual orientation health disparity after controlling for socio-demographic covariates, the inclusion of health behaviors yielded a 18 % decrease among men and a 10–17 % decrease among women in the association between sexual orientation and fair/poor self-rated health. Subsequently, exposure to minority stressors yielded a 14–25 % decrease in the association between sexual orientation and fair/poor self-rated health.

**Table 3:** Sexual orientation disparities in self-rated health by sex and age

Poor/fair self-rated health									
	%	Model 1		Model 2		Model 3		Model 4	
		OR (95 % CI)		OR (95 % CI)		OR (95 % CI)		OR (95 % CI)	
Men									
16–25 years (*n* = 3673)									
Heterosexual	11.7	1		1		1		1	
Gay or bisexual	26.9	2.90***	(2.02–4.18)	3.00***	(2.08–4.32)	2.53***	(1.72–3.72)	2.23***	(1.50–3.32)
26–35 years (*n* = 5027)									
Heterosexual	16.9	1		1		1		1	
Gay or bisexual	17.7	1.12	(0.71–1.76)	0.95	(0.60–1.52)	0.82	(0.51–1.32)	0.71	(0.44–1.15)
36–45 years (*n* = 12 400)									
Heterosexual	19.7	1		1		1		1	
Gay or bisexual	32.2	2.07***	(1.46–2.94)	1.50*	(1.05–2.16)	1.29	(0.88–1.89)	1.26	(0.85–1.88)
46–84 years (*n* = 35,254)									
Heterosexual	33.2	1		1		1		1	
Gay or bisexual	37.1	1.17	(0.90–1.54)	0.98	(0.74–1.30)	0.98	(0.73–1.31)	0.96	(0.72–1.30)

Women									
16–25 years (*n* = 4767)									
Heterosexual	16.8	1		1		1		1	
Lesbian or bisexual	35.2	2.68***	(2.02–3.56)	2.54***	(1.91–3.38)	2.27***	(1.69–3.06)	1.89***	(1.38–2.58)
26–35 years (*n* = 7158)									
Heterosexual	18.4	1		1		1		1	
Lesbian or bisexual	35.1	2.40***	(1.77–3.27)	2.24***	(1.64–3.06)	2.14***	(1.55–2.94)	1.98***	(1.42–2.75)
36–45 years (*n* = 16,957)									
Heterosexual	22.4	1		1		1		1	
Lesbian or bisexual	32.3	1.70**	(1.18–2.47)	1.44	(0.98–2.10)	1.45	(0.98–2.14)	1.46	(0.98–2.18)
46–84 years (*n* = 42,256)									
Heterosexual	37.1	1		1		1		1	
Lesbian or bisexual	42.3	1.24	(0.87–1.77)	1.15	(0.80–1.65)	1.14	(0.78–1.66)	1.14	(0.77–1.68)

* Significant at *p* < 0.05; ** significant at *p* < 0.01; *** significant at *p* < 0.001

*Model 1* unadjusted analysis. *Model 2* analyses are adjusted for income, ethnicity, household status, and living in urban or rural communities. *Model 3* analyses are adjusted for income, ethnicity, household status, and living in urban or rural communities, use of alcohol, tobacco, physical inactivity, and BMI. *Model 4* analyses are adjusted for income, ethnicity, household status, and living in urban or rural communities, use of alcohol, tobacco, physical inactivity, BMI, discrimination, victimization, and threats of violence. All models take into account sample weights

### Physical symptoms and conditions

In unadjusted analyses, gay/bisexual men and lesbian/bisexual women reported more physical symptoms and conditions than heterosexuals, and the differences were larger in the younger age groups (Table 4). The multivariate analyses showed a similar pattern as with self-reported general health. In the fully adjusted models, no sexual orientation differences in physical symptoms and conditions were observed among men older than 25 years and women ages 46 years and older. The disparities were reduced with the inclusion of covariates and became non-significant among men above 25 years of age, and among women above 45 years of age.

**Table 4:** Association between sexual orientation and self-reported physical symptoms and conditions by sex and age

Number of physical symptoms and conditions									
	M (SD)	Model 1		Model 2		Model 3		Model 4	
		Exp. β^a^ (95 % CI)		Exp. β^a^ (95 % CI)		Exp. β^a^ (95 % CI)		Exp. β^a^ (95 % CI)	
Men									
16–25 years (*n* = 3673)									
Heterosexual	2.2 (1.9)	1		1		1		1	
Gay or bisexual	3.4 (2.4)	1.26***	(1.19–1.34)	1.26***	(1.19–1.35)	1.22***	(1.14–1.30)	1.18***	(1.10–1.26)
26–35 years (*n* = 5027)									
Heterosexual	2.6 (2.1)	1		1		1		1	
Gay or bisexual	3.1 (2.2)	1.11**	(1.03–1.20)	1.09*	(1.01–1.18)	1.08	(0.99–1.17)	1.06	(0.98–1.15)
36–45 years (*n* = 12 400)									
Heterosexual	2.9 (2.2)	1		1		1		1	
Gay or bisexual	3.7 (3.0)	1.13***	(1.06–1.21)	1.08*	(1.01–1.16)	1.06	(0.99–1.14)	1.04	(0.97–1.11)
46–84 years (*n* = 35,254)									
Heterosexual	3.8 (2.5)	1		1		1		1	
Gay or bisexual	4.4 (2.8)	1.09**	(1.03–1.14)	1.06*	(1.00–1.11)	1.05	(1.00–1.10)	1.05	(1.00–1.10)

Women									
16–25 years (*n* = 4767)									
Heterosexual	3.2 (2.2)	1		1		1		1	
Lesbian or bisexual	4.4 (2.3)	1.19***	(1.12–1.25)	1.18***	(1.11–1.24)	1.16***	(1.09–1.22)	1.11***	(1.04–1.18)
26–35 years (*n* = 7158)									
Heterosexual	3.4 (2.3)	1		1		1		1	
Lesbian or bisexual	4.4 (2.4)	1.17***	(1.11–1.23)	1.15***	(1.09–1.22)	1.14***	(1.07–1.20)	1.11***	(1.05–1.18)
36–45 years (*n* = 16,957)									
Heterosexual	3.7 (2.4)	1		1		1		1	
Lesbian or bisexual	4.3 (2.5)	1.11**	(1.04–1.18)	1.07*	(1.00–1.14)	1.09*	(1.01–1.15)	1.07	(1.00–1.15)
46–84 years (*n* = 42,256)									
Heterosexual	4.7 (2.7)	1		1		1		1	
Lesbian or bisexual	5.0 (2.7)	1.06*	(1.00–1.13)	1.05	(0.99–1.12)	1.03	(0.97–1.10)	1.02	(0.96–1.09)

*Model 1* unadjusted analysis. *Model 2* analyses are adjusted for income, ethnicity, household status, and living in urban or rural communities. *Model 3* analyses are adjusted for income, ethnicity, household status, and living in urban or rural communities, use of alcohol, tobacco, physical inactivity, and BMI. *Model 4* analyses are adjusted for income, ethnicity, household status, and living in urban or rural communities, use of alcohol, tobacco, physical inactivity, BMI, discrimination, victimization, and threats of violence. All models take into account sample weights

* Significant at *p* < 0.05; ** significant at *p* < 0.01; *** significant at *p* < 0.001

^a^ Exponential beta coefficient

## Discussion

Although several recent studies have documented sexual orientation disparities in physical health [6], there is a paucity of research exploring whether these disparities differ across the lifespan. The few studies that have examined age differences have found that sexual orientation disparities are present among both younger and older individuals [7, 20], consistent with cumulative stress theories. In contrast to these initial studies, our results indicated that age is an important effect modifier of sexual orientation disparities in physical health. Using data from a large, nationally representative sample of individuals between 16 and 84, we show that sexual orientation disparities in self-rated health and in physical health symptoms/conditions are largest among adolescents and young adults, and smallest among the oldest age groups. These results mostly support a developmental model proposing larger health disparities among younger individuals due to elevated age-specific minority stress experiences.

One exception to this pattern was elevated health disparities between sexual minority and heterosexual early middle-aged men (36–45 years). However, this disparity was eliminated once health behaviors were statistically controlled, and thus could potentially be explained by elevated detrimental health behaviors among gay and bisexual men in this age group. The pattern of larger health disparities among younger LGB individuals was also found for reports of various physical health symptoms and conditions, with results showing large disparities in the adolescent and young adult group and smaller disparities among adult and older adult sexual minorities. These disparities were attenuated slightly after adjustments for potential confounding variables such as demographics and socioeconomic factors, health behaviors, and minority stress factors. The reduction of sexual orientation health differences in the analyses when health behaviors and BMI were included indicates important disadvantages in the area of detrimental health behaviors and body weight among LGB individuals. These findings lend support for the inclusion of health behaviors in minority stress models when applied to physical health disparities.

A recent study from the US based on a smaller population-based sample showed similar results to ours, with the largest disparities in self-reported health observed among younger adults (18–29 years) and the smallest among adults aged 50–59 years [33]. Although that study provided important insights, it was not large enough to analyze gender separately. Our ability to conduct gender-stratified models in this study revealed some notable sex differences in health disparities across age groups. In particular, the proportion of sexual minority women reporting poor health was essentially stable (slightly above 30 %) until age 45, in contrast to the pattern among sexual minority men who reported the lowest level of poor health in early adulthood (26–35 years), and no sexual orientation difference in health in the fully adjusted models among those older than 25 years.

Risk factors for ill health, including experiences of minority stress and health-risk behaviors, were more prevalent among sexual minorities than heterosexuals. Sexual minority individuals were much more likely to report perceived discrimination, victimization, and threats of violence as compared to heterosexuals. These differences were most pronounced in the younger age group, and the disparity in victimization was generally strongest among younger sexual minority women and middle-age gay and bisexual men. The elevated reports of exposure to perceived discrimination, victimization, and threats of violence among sexual minorities partially explained the sexual orientation physical health disparities. Thus, our findings indicate that differences in physical health can partially be explained by higher exposure to minority stress among the sexual minority group. Both the physical health disparities and the disparity explained by elevated reports of exposure to stressors were largest in the younger age groups.

These results are consistent with previous studies reporting disproportionate experiences of adverse self-reported health among LGB individuals compared to heterosexuals [11, 34–36]. Previous studies have also reported an increased prevalence of the specific physical health symptoms and conditions included in this study, for example, neck pain [9], intestinal problems [9, 22], headache [9, 22], urinary incontinence [9], asthma [11, 20, 22, 37, 38], back pain [22], and fatigue [22].

In addition to providing support for the minority stress model of physical health [6], our results also lend new support to an age-based, lifespan model of sexual orientation disparities in physical health, whereby LGB individuals report greater exposure to stressors than heterosexuals earlier in the lifespan, with these stressors generally decreasing across age groups. These results support a developmental model of minority stress and physical health among LGB individuals whereby the stressors of navigating a stigmatized public identity are greater in adolescence and young adulthood and are associated with poorer physical health than in later years [16]. In contrast, these results are not consistent with a lifespan accumulation effect of stigma and physical health found for other disadvantaged social groups [17–19], as the largest physical health and minority stress disparities were found among adolescents and young adults, rather than older adults. However, because the data are cross-sectional, causal conclusions cannot be made, and alternative explanations to these results cannot be excluded.

### Limitations

Several features inherent to self-report population-based health surveys somewhat limit our study. Given that the variables of interest in the present study were asked of both sexual minority as well as heterosexual respondents, we are unable to examine sexual minority-specific processes potentially relevant to health (e.g., internalized homophobia, status-based rejection sensitivity, sexual orientation concealment). However, by investigating health determinants reported by both groups, we were able to determine whether sexual orientation disparities in measured determinants account for sexual orientation disparities in physical health outcomes. Further, given that data were collected cross-sectionally at each assessment point, we are unable to establish the causal direction of effects and unable to determine the influence of cohort effects, such as improved laws, policies, and social attitudes surrounding sexual minorities over time, despite the relevance of social change to any life course minority stress model of sexual minority physical health [6]. The pooled data from several years also has limitations in that a small subset of individuals might have been included in more than one data collection, and the circumstances for LGB individuals might have changed somewhat over time. However, we consider it unlikely that these limitations influenced our overall conclusions. Further, self-report measures of stressful experiences may be confounded with health status, which might yield biased estimates of the association between stress and health [39]. It is also possible that some of the age differences in health identified in the study are influenced by selection factors, such as increased mortality rates in the LGB group (i.e., survivorship bias) and lower likelihood of reporting LGB status in older age groups. However, prior population-based studies have found limited evidence for overall differential mortality risk between sexual minorities and heterosexuals, which makes such survivorship bias unlikely [40, 41]. Recent research by Hatzenbuehler and colleagues has shown premature mortality among sexual minorities in high stigma communities [42], but in that study few communities were characterized by high stigma. The sample used in the current study consists of a national sample from Sweden, a country with a comparably low level of structural stigma and high level of acceptance of sexual minorities. Poorer health among gay and bisexual men could also have been influenced by elevated rates of HIV infections in this group, but information regarding HIV status was not available for the current sample. However, the median age of HIV diagnoses among men who have sex with men in Sweden is 34 years, with a comparably high proportion of this group (45 %) receiving their diagnosis early (within 6 months of infection) [43]. Thus, given that the large sexual orientation health disparities for men were found in the youngest age group (age 16–25 years) in this study, we consider it unlikely that our inability to control for HIV status in the analyses influenced our overall conclusions. Lastly, given the relatively small number of LGB respondents in the oldest age groups (i.e., older than 65–84), we had to combine respondents in ages 46–84, potentially obscuring important subgroup differences related to age.

We interpret our current findings as supporting an age effect, since we identify elevated sexual orientation-based health differences in the younger age groups. However, the lack of sexual orientation-based health differences in the older age groups does not exclude a cohort effect even though the earlier cohorts (i.e., the older respondents) should be less healthy because of exposure to minority stressors during a longer time period. The lack of such finding in our current study could potentially be due to a healthy survivor effect, which we are unable to assess with the present data. Nevertheless, results of this study suggest the importance of follow-up studies that utilize diverse designs, such as age-period-cohort methods, and measurement approaches such as objective measures of stress and health, to further confirm the lifespan model proposed here. Such a study would clarify the relative importance of age effects versus cohort effects in understanding sexual orientation health differences.

### Strengths

The study also has a number of strengths, including the fact that this is the largest dataset with information on sexual orientation in Sweden and it uses a nationally representative sample from the population. Many studies of sexual orientation health disparities rely on nonrandom samples, which limit generalizability of the findings [6]. The sample size also enabled us to stratify analyses by both gender and age groups, which revealed important gender and age differences in health disparities and risk factors for health that could not have been found in studies with smaller groups of LGB individuals or samples limited to a particular gender or age group.

## Conclusions

This study reveals novel information on age patterning indicating that physical health disparities based on sexual orientation are largest among adolescents and young adults, and smallest in the oldest age groups. Our findings indicated that differences in physical health were partially explained by higher exposure to minority stress and more frequent health detrimental behaviors. Knowledge from this study regarding age group differences in sexual orientation physical health disparities and determinants of those disparities can facilitate further tests of life course models of sexual minority physical health and the development of targeted psychosocial interventions to improve the health of LGB individuals—a clear public health goal [1].
